# Influence of dairy products consumption on oral cancer risk: A meta-analysis

**DOI:** 10.34172/joddd.2023.36851

**Published:** 2023-04-03

**Authors:** Alberto Rodriguez-Archilla, Marina Gomez-Fernandez

**Affiliations:** Department of Stomatology, Oral Medicine Unit. Faculty of Dentistry. University of Granada, Granada, Spain

**Keywords:** Butter, Cheese, Dairy products, Milk, Mouth neoplasms, Yogurt

## Abstract

**Background.:**

The role of dairy product consumption on oral cancer risk is not yet fully clarified. Some studies have observed an inverse association between dairy consumption and oral cancer risk. This study aimed to determine the influence of dairy product consumption (milk, cheese, yogurt, butter) on oral cancer risk.

**Methods.:**

A search for studies on dairy products and oral cancer was conducted in the following databases: PubMed (MEDLINE, Cochrane Library), Web of Science (WoS), and Scopus. The estimation of the odds ratio (OR) effect was performed with the generic inverse variance method using the logarithm of the effect with the standard error (SE) and 95% confidence intervals.

**Results.:**

Twenty-one studies with 59271 participants (8,300 oral cancer patients and 50971 controls) were included in this meta-analysis. All dairy products significantly reduced oral cancer risk except butter (*P*=0.16). Milk intake reduced oral cancer risk by 27% (OR: 0.73; *P*<0.001); yogurt consumption by 25% (OR: 0.75; *P*<0.001), and cheese consumption by 21% (OR:0.79; *P*<0.01).

**Conclusion.:**

Regular consumption of dairy products reduces oral cancer risk between 21% and 27%.

## Introduction

Oral cancer is the eighth most common cancer in men worldwide, being oral squamous cell carcinoma (OSCC) as the most common histological type (90% of cases). The main oral cancer risk factors are tobacco consumption, alcohol intake, betel nut use, genetic factors, HPV infection, chronic oral mucosal trauma together with poor oral hygiene, and diet.^1^ Dietary factors have been related to oral cancer risk, especially the consumption of tea or coffee, fruits, vegetables, or meat. However, there are few studies on dairy product consumption and oral cancer risk with inconclusive results. The influence of dairy product consumption on oral cancer risk is still not fully understood.^2^ Apart from insulin-like growth factor (IGF) and calcium, which are risk factors for prostate cancer; milk lipids and fatty acids (linoleic acid, butyric acid, phospholipids, and sphingolipids), are probably beneficial agents against cancer. Milk contains high-quality protein, which can improve immunity and promote the body to recover health. Drinking milk and consuming dairy products may protect against oral cancer.^3^ Some studies have found an inverse association between dairy product consumption and oral cancer risk.^4^ This study aimed to determine the influence of the consumption of dairy products (milk, cheese, yogurt, butter) on oral cancer risk.

## Methods

 All research steps (search, study selection, and data extraction) were achieved independently by both authors (ARA and MGF). Discrepancies in article selection were resolved by consensus.

###  Search strategy

A search for studies on the influence of dairy products (milk, cheese, yogurt, butter) consumption on oral cancer risk up to October 2022 was performed in the following databases: PubMed (MEDLINE, Cochrane Library), Web of Science (WoS), and Scopus. The search strategies in each database using a combination of Medical Subjects Headings (MeSH) and free-text terms are shown in Table 1. The inclusion criteria were as follows: (a) all types of articles related to our purpose, (b) articles without relevant risk of bias (score ≥ 6 stars on the Newcastle-Ottawa methodological quality assessment scale),^5^ and (c) articles written in any language and with no restrictions on publication date. The exclusion criteria were: (a) articles with no full-text availability, (b) articles with no clinical data, and (c) studies with non-usable data.

**Table 1:** Search strategies for the three databases

**Database**	**#**	**Search strategy**	**Results**
PubMed	#1	“dairy products”[MeSH Terms] OR “milk”[All Fields]	173,518
	#2	“mouth neoplasms”[MeSH Terms] OR “oral cancer”[All Fields]	80,414
	#3	#1 AND #2	65
Web of Science (WoS)	#4	("dairy products"[Topic] OR "milk"[Topic])	433,524
	#5	("mouth neoplasms"[Topic] OR "oral cancer"[Topic])	51,243
	#6	#4 AND #5	73
Scopus	#7	TITLE-ABS-KEY ("dairy products" OR "milk")	256,910
	#8	TITLE-ABS-KEY ("mouth neoplasms" OR "oral cancer")	46,428
	#9	#7 AND #8	59

###  Assessment of methodological quality

 The methodological quality of the articles was screened using the Newcastle-Ottawa Scale (NOS) methodological quality assessment scale composed of eight items that evaluate three dimensions (selection, comparability, and exposure). Considering the score obtained, the studies are classified as high quality ( ≥ 7 stars), moderate quality (4-6 stars), and low quality (1-3 stars).

###  Statistic analysis

Data were meta-analyzed with the RevMan 5.4 program (The Cochrane Collaboration, Oxford, UK). The estimation of the odds ratio (OR) effect was conducted with the generic inverse variance method, using the logarithm of the effect with the standard error (SE) and 95% confidence intervals (95% CI). Heterogeneity was determined according to the Higgins statistic (I^2^). In cases of high heterogeneity (I^2^ > 50%), the random-effects model was applied. The minimum level of significance was set at *P* < 0.05.

## Results

###  Study selection

**Figure 1:**
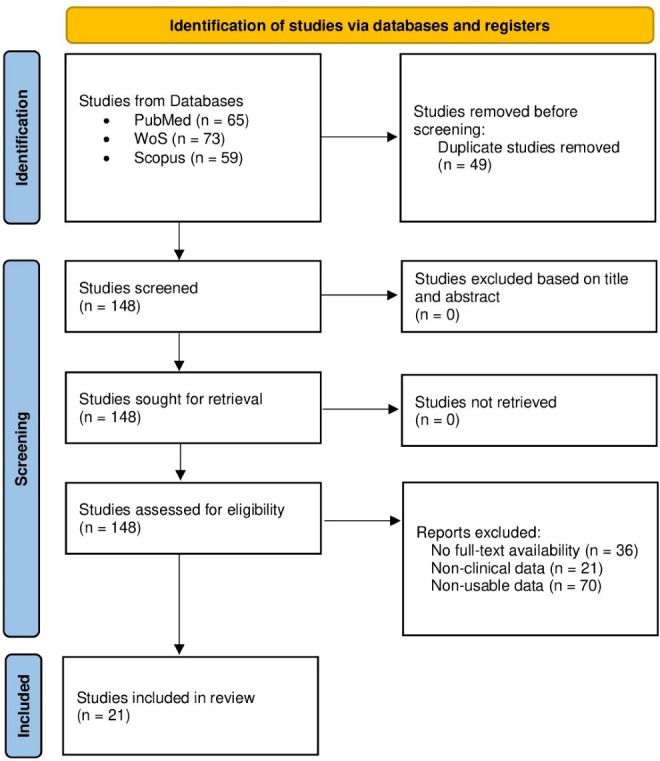
Study selection flowchart

The search found 197 articles (65 in PubMed, 73 in WoS, and 59 in Scopus) between the years 1977 and 2021, 49 of them duplicates, leaving 148 articles for eligibility. 127 articles were excluded due to: (a) articles with no full-text availability (n = 36), (b) articles without clinical data (n = 21), and (c) studies with non-usable data (n = 70). Finally, 21 studies were included in this meta-analysis (Figure 1).

Table 2 presents the main descriptive characteristics and the methodological quality according to the NOS scale of the twenty-one studies^6-26^ included in this meta-analysis. A total of 59 271 participants (8300 oral cancer patients and 50 971 controls) were considered. By gender, 23 551 were males (41.2%), and 33 384 were females (58.8%). The studies were conducted in the following countries: Italy (3 studies), the United States of America (3 studies), Brazil (3 studies), India (2 studies), China (2 studies), Switzerland (2 studies), Japan (1 study), Cuba (1 study), Uruguay (1 study), Greece (1 study), Spain (1 study), and Poland (1 study). Considering the NOS quality scale, four articles (19.0%) had 6 stars, fourteen articles (66.7%) got 7 stars, and three articles (14.3%) reached 8 stars.

**Table 2:** Characteristics and methodological quality evaluation of the twenty-one articles included in this meta-analysis

**Study, year**	**Country**	**Study population**	**Dairy product**	**Other parameters analyzed **	**NOS**
Notani, 1987^6^	India	278 OC (na, na; na) 215 CS (na, na; na)	Milk	Tobacco, food groups	7
La Vecchia, 1991^7^	Italy	105 OC (89 M, 16 F; 37-74 y) 1169 CS (875 M, 294 F; 21-74 y)	Milk, cheese, butter	Age, gender, tobacco	6
De Stefani, 1994^8^	Uruguay	246 OC (246 M, 0 F; 40-89 y) 253 CS (253 M, 0 F; 40-89 y)	Milk	Age, gender, tobacco, alcohol, food groups	6
Takezaki, 1996^9^	Japan	266 OC (189 M, 77 F; 20-89 y) 36527 CS (9858 M, 26669 F; 20-89 y)	Milk	Age, gender, tobacco, alcohol, food groups	7
Levi, 1998^10^	Switzerland	156 OC (126 M, 30 F; 26-72 y) 284 CS (246 M, 38 F; 23-74 y)	Milk	Age, gender, food groups	7
Franceschi, 1999^11^	Italy	271 OC (219 M, 52 F; 22-77 y) 1491 CS (1008 M, 483 F; 20-78 y)	Milk, cheese, butter	Age, gender, food groups	7
Morse, 2000^12^	USA	87 OC (51 M, 36 F; 20-79 y) 87 CS (51 M, 36 F; 20-79 y)	Milk	Age, gender, food groups	6
Garrote, 2001^13^	Cuba	200 OC (143 M, 57 F; 28-91 y) 200 CS (136 M, 64 F; 25-88 y)	Milk, cheese,	Age, gender, ethnic group, education, occupation, tobacco, alcohol, food groups	7
Petridou, 2002^14^	Greece	106 OC (65 M, 41 F; na) 106 CS (65 M, 41 F; na)	Milk	Age, gender, education, tobacco, alcohol, BMI, food groups	7
Lissowska, 2003^15^	Poland	122 OC (78 M, 44 F; na) 124 CS (72 M, 52 F; na)	Milk, cheese, yogurt	Age, gender, tobacco, alcohol, genderual habits, food groups	8
Rajkumar, 2003^16^	India	591 OC (309 M, 282 F; 18-87 y) 582 CS (292 M, 290 F; 18-80 y)	Milk, cheese, yogurt	Age, gender, education, tobacco, alcohol, BMI, food groups	7
Sánchez, 2003^17^	Spain	375 OC (304 M, 71F; 20-91 y) 375 CS (304 M, 71F; 20-87 y)	Milk, cheese, yogurt	Age, gender, occupation, tobacco, alcohol, food groups	8
Toporcov, 2004^18^	Brazil	70 OC (50 M, 20 F; 34-77 y) 70 CS (50 M, 20 F; 35-81 y)	Milk, cheese, butter	Age, gender, food groups	6
Gallus, 2006^19^	Italy	598 OC (512 M, 86 F; na) 1491 CS (1008 M, 483 F; na)	Milk, cheese, yogurt	Age, gender, food groups	7
Kreimer, 2006^20^	USA	1670 OC (na, na; na) 173 CS (na, na; na)	Milk, cheese,	Tobacco, alcohol, BMI, food groups	7
Marchioni, 2007^21^	Brazil	366 OC (310 M, 56 F; na) 469 CS (370 M, 99 F; na)	Milk, cheese, yogurt, butter	Age, gender, education, tobacco, alcohol, food groups	7
Sapkota, 2008^22^	USA	378 OC (331 M, 47 F; 45-74y) 916 CS (736 M, 180 F; 45-74y)	Milk, cheese, yogurt	Age, gender, education, tobacco, alcohol, food groups	8
Toporcov, 2012^23^	Brazil	296 OC (230 M, 66 F; na) 296 CS (230 M, 66 F; na)	Milk, cheese, yogurt, butter	Age, gender, tobacco, alcohol, food groups	7
Bravi, 2013^24^	Switzerland	768 OC (593 M, 175 F; 22-79 y) 2078 CS (1368 M, 710 F; 19-79 y)	Milk, cheese, yogurt	Age, gender, education, BMI, food groups	7
Chen, 2017^25^	China	421 OC (105 M, 316 F; 20-91 y) 1398 CS (402 M, 996 F; 20-89 y)	Milk	Age, gender, education, BMI, food groups	7
Chen, 2017^26^	China	930 OC (588 M, 342 F; 20-80 y) 2667 CS (1689 M, 978 F; 20-80 y)	Milk	Age, gender, education, tobacco, alcohol, BMI, food groups	7

OC: oral cancer patients; CS: controls without cancer; M: male; F: female; y: age range in years; na: data not available; BMI: body mass index; NOS: Newcastle-Ottawa scale.

###  Milk

**Figure 2:**
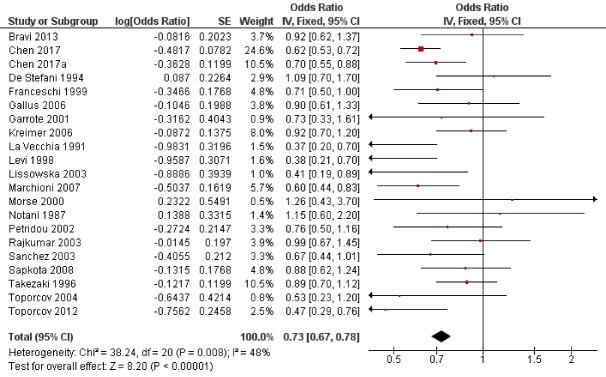
Study data and forest plot graph for the influence of milk intake on oral cancer risk. SE: standard error

Twenty-one studies^6-26^ examined the possible influence of milk intake on oral cancer risk (Figure 2). Regular milk consumption reduced by 27% the oral cancer risk by with a highly statistically significant association (OR = 0.73; 95% CI: 0.67 to 0.78; *P* < 0.001).

###  Cheese

**Figure 3:**
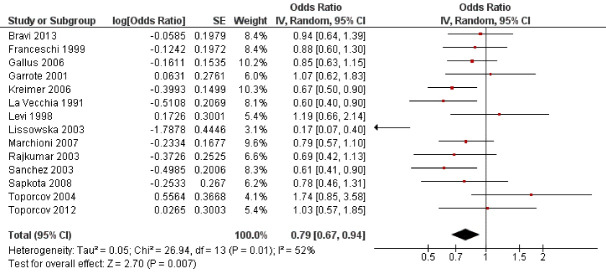
Study data and forest plot graph for the influence of cheese consumption on oral cancer risk. SE: standard error

Fourteen studies^7,10,11,13,15-24^ analyzed the possible influence of cheese consumption on the probability of oral cancer (Figure 3), finding that cheese decreased by 21% oral cancer risk. After statistical analysis, a highly significant relationship was found (OR = 0.79; 95% CI: 0.67 to 0.94; *P* < 0.001).

###  Other dairy products (yogurt, butter)

**Figure 4:**
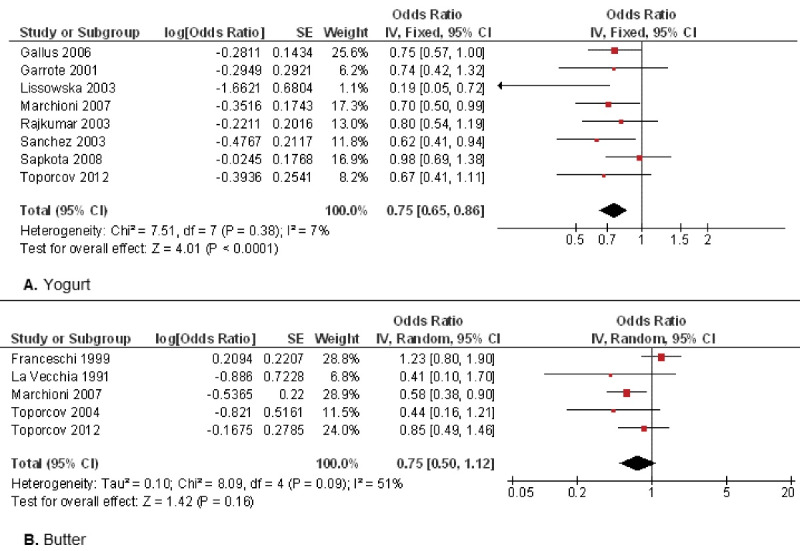
Study data and forest plot graphs for the influence of yogurt consumption (A) or butter consumption (B) on oral cancer risk. SE: standard error

Figure 4 shows the evaluation of other dairy products such as yogurt or butter. Eight studies^13,15-17,19,21-23^ evaluated yogurt consumption (Figure 4A), confirming that it reduced by 25% the probability of oral cancer. Statistical analysis confirmed highly significant differences (OR = 0.75; 95% CI: 0.65 to 0.86; *P* < 0.001). Five studies^7,11,18,21,23^ focused on butter consumption as a possible oral cancer risk factor (Figure 4B). Although butter consumption appears to reduce the oral cancer risk, the results were not statistically significant (OR = 0.75; 95% CI: 0.50 to 1.12; *P* = 0.16).

## Discussion

 Data from 21 studies about the influence of dairy product intake (milk, cheese, yogurt, butter) on oral cancer risk were included in the present meta-analysis.

The role of regular dairy products intake on cancer risk is controversial, with apparently conflicting results depending on the location of cancer. Regarding oral cancer, some studies maintain that dairy products induce an increase in oral cancer risk, while others affirm that dairy consumption has a protective effect.^27^

Many studies have confirmed the relationship between varied food components and oral cancer. The intake of red meat and dairy products has been linked to higher levels of saturated fats. The frequent intake of saturated fats from dairy products has been positively associated with higher oral cancer risk, especially in people that consume significant amounts of cakes, cheese, or ice creambars.^28^ Although some studies have related dairy products to head and neck neoplasms, the real influence of these foods on the genetic transcription factors expression in oral cancers has not been established so far.^29^

Using dairy products to improve oral health may have several additional health effects. Probiotic dairy products may inhibit *Candida* growth in the oral environment. It is thought that dairy products may change the saliva composition, such as the salivary immunoglobulins and mucins. Probiotic dairy products may be a promising choice to improve oral health, including the reduction of *Candida* superinfection in oral cancers.^30^

In this study, regular milk consumption reduced by 27% the oral cancer risk with a highly statistically significant relationship (*P* < 0.001). Of the twenty-one studies that analyzed the role of milk consumption on oral cancer risk, eighteen^7,9,10,11,13-26^ agreed that milk had a protective effect on cancer risk; while the remaining three^6,8,12^ did not observe it. A dietary score to assess the influence of the consumption of different foods (vegetables, seafood, milk, and other dairy products) on oral cancer risk has been proposed. People who ate dairy products regularly not only had lower rates of oral cancer than those who did not but the greater the amount of dairy intake, the more this risk decreased.^26^ In contrast, a study conducted in the United States found the opposite, showing that dairy product consumption was associated with the development of epithelial dysplasia, which is associated with increased oral cancer risk.^12^

In the present study, cheese consumption reduced by 21% oral cancer with a highly statistically significant association (*P* < 0.001). Of the fourteen studies that investigated cheese consumption, ten of them^7,11,15-17,19-22,24^ confirmed this lower risk of cancer-related to this food, compared to the four studies^10,13,18,23^ who disagreed and did not observe this risk reduction. Studies carried out on cheese consumption and oral cancer risk showed inconsistent results, some establishing a positive association and others an inverse association. Cheese appears to have a protective effect against oral cancer, due to its high content of conjugated linoleic acid, which has immunostimulatory and anticancer properties.^22^ Others do not observe this protective effect of cheese, associating its high consumption with a significant caloric intake. The intake of foods rich in calories such as cheese, other dairy products, bread, potatoes, eggs, or alcoholic beverages could explain the apparent potentiating effect of oral cancer.^18^ However, moderate cheese consumption has a marked protective effect on oral cancer risk.^15^

In this meta-analysis, yogurt intake reduced by 25% the oral cancer risk with highly statistically significant differences (*P* < 0.001). All the studies^13,15-17,19,21-23^ that analyzed this food corroborated the protective effect of yogurt consumption on oral cancer. The population of Western countries has a low consumption of milk and yogurt, but considerably high consumption of cheese. The consumption of dairy products, including yogurt, does not show a consistent association with upper respiratory tract cancers, with conflicting results regarding its true influence on them. However, in the case of oral cancer, yogurt was the dairy derivative with the most favorable effect on oral cancer risk.^19^ A study conducted in Brazil confirms the protective effect of consuming yogurt and other dairy products on the risk of oral cancer. Regular consumers of these dairy products, in adequate amounts, benefited from this protective effect, decreasing the incidence of oral cancer.^21^ However, this effect was reversed when the intake of these foods occurred in large amounts, probably due to the large increase in calories ingested.^22^

In the present study, butter consumption seemed to decrease oral cancer risk, although the results were not statistically significant (*P* = 0.16). Of the five studies that evaluated butter, four of them^18,20,21,23^ found this protective effect of butter on oral cancer; while a single study^11^ found an increased oral cancer risk associated with butter consumption. The true influence of butter intake on oral cancer risk is not well established and the results are controversially requiring further investigation.^23^ However, butter consumption is a source of vitamin A and carotenoids, micronutrients with a protective effect against potentially malignant lesions and various cancers, including oral cancer. This effect is enhanced when it is consumed unprocessed as a complement to other foods.^18^ A multicenter study carried out in several countries on the role of dietary habits in oral cancer risk revealed that the consumption of processed meats, butter, and alcoholic beverages were the most relevant risk factors for the appearance of oral cancer. On the contrary, the consumption of fish, raw vegetables, and oil were the most important protective factors, significantly reducing the oral cancer risk occurrence.^11^

## Limitations of the study

 This study presents some limitations. Could not distinguish between different types of milk (whole, part-skim, skim, lactose-free, etc.) and their effect on oral cancer risk. Nor has it been possible to assess the amount of these dairy products consumed and their real influence on oral cancer risk. Finally, although the observed heterogeneity in some comparisons was not very high, the results must be interpreted with caution. New cohort studies with longer follow-up times are required to further explore the association between dairy product consumption and oral cancer risk.

## Conclusion

In this meta-analysis, all dairy products significantly reduced oral cancer risk, except butter (*P* = 0.16). Milk intake reduced oral cancer risk by 27%, yogurt consumption by 25%, and cheese consumption by 21%.

## Competing Interests

 None.

## Ethical Approval

 Not applicable.

## Funding

 None.
